# Extraskeletal Ewing sarcoma primarily arising in the stomach and omentum in children: a case series

**DOI:** 10.1093/jscr/rjag581

**Published:** 2026-07-13

**Authors:** Isabel M Kiko, Vineet Narayanan, Bader Kiwan, Benson Li, Autumn Pak, Matthew Keisling, Justin T Huntington

**Affiliations:** Department of General Surgery, Summa Health Systems, Akron, OH, United States; College of Medicine, Northeast Ohio Medical University, Rootstown, OH, United States; Department of General Surgery, St. Elizabeth’s Hospital, Youngstown, OH, United States; Department of General Surgery, Summa Health Systems, Akron, OH, United States; Department of General Surgery, Summa Health Systems, Akron, OH, United States; Department of Pathology and Laboratory Medicine, Akron Children’s Hospital, Akron, OH, United States; Department of Pediatric Surgery, Akron Children’s Hospital, Akron, OH, United States

**Keywords:** Ewing sarcoma, extraskeletal Ewing sarcoma, intra-abdominal sarcomatosis, radiographic-pathologic dissonance, pediatric surgical oncology, case series

## Abstract

Primary visceral extraskeletal Ewing sarcoma is exceedingly rare; gastric and omental primaries pose diagnostic challenges by mimicking benign gastrointestinal conditions. We present a 17-year-old with a gastric mass and a 12-year-old with an omental primary, mismanaged as iron deficiency anemia and functional constipation, respectively. Diagnoses were confirmed via imaging, endoscopy, and histopathology with EWSR1::ERG and EWSR1::FLI1 fusion detection. Both received neoadjuvant chemotherapy for cytoreduction and organ-sparing surgery. The first underwent wedge gastrectomy with positive serosal margins, lymphovascular invasion, and nodal involvement necessitating adjuvant therapy. The second, presenting with peritoneal sarcomatosis and bone marrow metastasis, underwent en bloc enterectomy, achieving 5-year disease-free survival despite toxicities requiring early discontinuation. These cases underscore radiographic-pathologic dissonance, wherein imaging regression may not reflect biological clearance, and high suspicion for small round blue cell tumors in refractory presentations. Multimodal integration of molecular diagnostics, induction chemotherapy, and aggressive cytoreduction optimizes visceral extraskeletal Ewing sarcoma outcomes.

## Introduction

Extraskeletal Ewing sarcomas (EES) constitute ~20%–25% of all Ewing family neoplasms, originating from mesenchymal soft tissue as opposed to medullary or cortical bone [1, 2]. In contrast to the male-skewed predominance observed in skeletal Ewing presentations, EES exhibits a marginal female predilection [3]. While EES most commonly manifests in the axial soft tissues or the pelvic girdle and limbs, primary infiltration of the alimentary canal represents an anomalous finding [2, 4].

In the most extensive clinicopathologic review of gastrointestinal Ewing sarcoma to date, Yin et al. analyzed 25 instances, of which the small bowel served as the primary site for the overwhelming majority (84%) of cases, while gastric localization was comparatively infrequent [5]. Ages ranged from 9 to 59 years, with no significant sex bias [5]. Notwithstanding these findings, cumulative longitudinal case data reveal that primary gastric Ewing sarcoma remains a diagnostic rarity, with fewer than 20 reported occurrences; these cases span a broad age demographic (14 to 66 years) and demonstrate a marked female preponderance [6, 7]. Even more exceptional is the primary omental variant, which is currently characterized only by sporadic, isolated case findings [8].

Patients with gastric EES typically present with non-specific abdominal complaints, including epigastric pain, early satiety, nausea, vomiting, gastrointestinal hemorrhage, symptomatic anemia, and, in advanced stages, gastric outlet obstruction [5–7]. Due to the variability in clinical exhibition, surgical approaches have ranged from laparoscopic wedge resection to distal, subtotal, or total gastrectomy, with some cases necessitating Roux-en-Y reconstruction or pancreaticoduodenectomy (Whipple procedure) [9, 10]. Standard management typically entails a multimodal strategy integrating neoadjuvant and/or adjuvant chemotherapy with surgical resection, in alignment with treatment paradigms for Ewing sarcoma at other body sites [3]. Despite aggressive treatment, gastrointestinal Ewing sarcoma carries a poor prognosis, with one series reporting a mortality rate of 63.2% at a median follow-up of 12 months [5].

By evaluating a series of pediatric gastric and omental Ewing sarcomas, this report seeks to explicate the symptomatology and diagnostic subtleties inherent to these rare malignancies, with the auxiliary intent of refining clinical suspicion and expediting the therapeutic timeline.

## Case series

### Case 1

A 17-year-old female presented ~1 year prior to definitive diagnosis with progressive exertional dyspnea and presyncope exacerbated by athletic participation. She was empirically treated with bronchodilator therapy for presumed exercise-induced bronchospasm with minimal symptomatic improvement. Subsequent laboratory findings revealed profound anemia (Hb < 6 g/dl), warranting hospital admission for packed red blood cell transfusion and comprehensive diagnostic workup. The patient denied gastrointestinal symptomatology or overt hemorrhage. Despite oral iron supplementation for suspected nutritional iron deficiency secondary to a vegetarian diet, her symptoms persisted and eventually progressed to include postprandial abdominal pain.

Transabdominal ultrasonography revealed diffuse, circumferential gastric mural thickening suggestive of an inflammatory or infiltrative process, prompting gastroenterology consultation. Given persistent microcytic anemia refractory to enteral iron replacement, bidirectional endoscopy was indicated to exclude occult gastrointestinal hemorrhage and characterize the gastric pathology. Esophagogastroduodenoscopy demonstrated multiple well-defined ulcerative lesions within the gastric mucosa and a conspicuous submucosal bulge. The overlying mucosa appeared endoscopically unremarkable, without evidence of friability or stigmata of recent hemorrhage, suggesting a lesion originating from the tunica muscularis or submucosal layers.

Contrast-enhanced computed tomography (CECT) of the chest, abdomen, and pelvis demonstrated a large gastric mass along the lesser curvature, accompanied by both endophytic and exophytic elements (Fig. 1a and b). Imaging raised concerns for potential hepatic involvement and prominent lymphadenopathy within the hepatogastric pars flaccida. An endoscopic ultrasound (EUS)-guided core biopsy of the gastric submucosal mass confirmed a definitive diagnosis of Ewing sarcoma. Further staging with positron emission tomography (PET) scan identified a hypermetabolic thyroid nodule; fine needle aspiration (FNA) biopsy confirmed papillary thyroid carcinoma. Germline genetic testing was negative for known familial oncologic syndromes but identified a heterozygous variant of unknown significance in the *ALK* gene.

**Figure 1:**
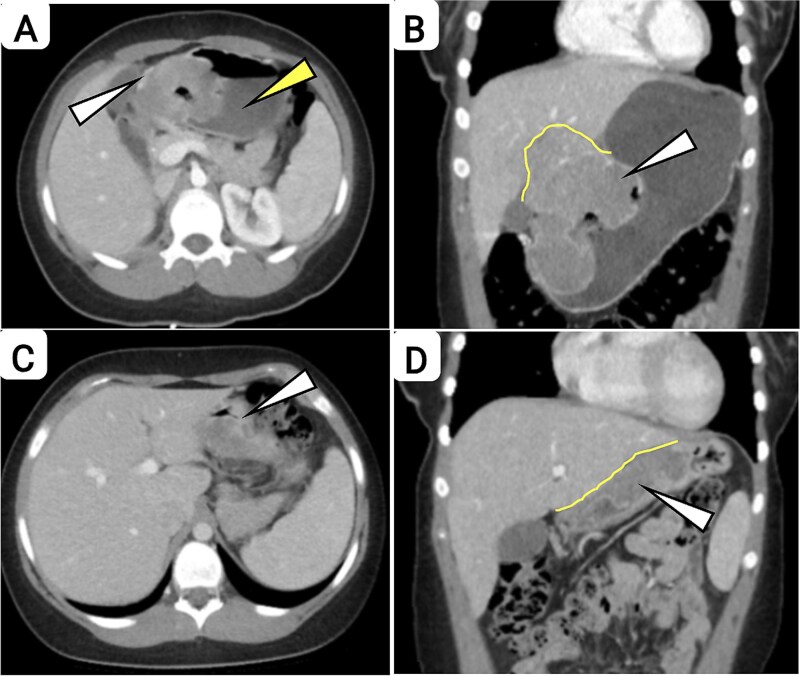
CECT of the abdomen and pelvis, demonstrating treatment response in Case 1. (a and b) Pre-treatment images demonstrate a voluminous, heterogeneously enhancing gastric neoplasm centered along the lesser curvature, exhibiting a complex growth pattern with both endophytic and exophytic components. (a) Axial image: The white arrow denotes the peripherally enhancing tumoral rim, and the yellow arrow delineates an internal hypoattenuating component consistent with liquefactive necrosis or cystic degenerative change. (b) Coronal image: The white arrow denotes the predominant exophytic neoplastic mass, while the yellow curved line delineates obliteration of the intervening fat plane with contiguous extension into the adjacent hepatic parenchyma, suggesting direct visceral invasion and resultant luminal compromise. (c and d) Post-treatment images obtained following neoadjuvant systemic therapy demonstrate a substantial partial radiographic response (RECIST criteria), characterized by marked interval reduction in the primary gastric mass and associated components. (c) Axial image: The white arrow demarcates residual hypoattenuating soft tissue proliferation persisting in the gastrohepatic ligament. (d) Coronal image: The white arrow denotes minimal residual soft tissue within the gastrohepatic ligament, and the yellow curved line delineates reconstitution of the fat plane interposed between the gastric wall and hepatic parenchyma, with no definitive evidence of persistent hepatic invasion. Interval regression of gastrohepatic lymphadenopathy is evident.

The patient was initiated on neoadjuvant chemotherapy per protocol AEWS1221, consisting of etoposide and ifosfamide with mesna. Post-treatment CT imaging demonstrated a significant reduction in primary gastric tumor burden with no radiographic evidence of hepatic metastases (Fig. 1c and d). Persistent but reduced gastrohepatic lymphadenopathy was noted. The patient was subsequently deemed an appropriate candidate for surgical intervention.

An exploratory laparotomy was performed via a left subcostal incision. Intraoperative assessment focused on the lesser curvature and gastrohepatic ligament. A single enlarged lymph node was excised for pathological evaluation. No gross hepatic involvement was observed. The primary gastric tumor was resected via wedge gastrectomy using a linear stapling device over a 38-French bougie to preserve luminal patency. Gross examination demonstrated a lobulated, exophytic gastric neoplasm with a tan-white fleshy appearance; serial sectioning revealed a solid mass with hemorrhagic degeneration and focal necrosis (Figs 2 and 3).

**Figure 2:**
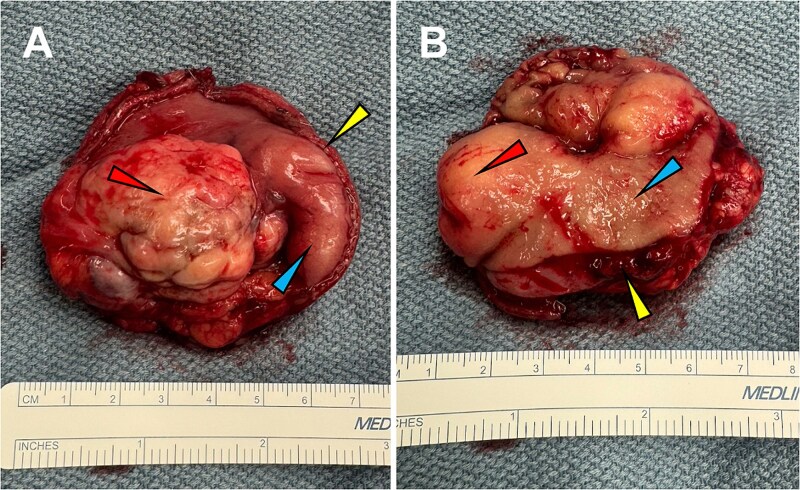
Gross surgical specimen following wedge gastrectomy, Case 1. A lobulated, exophytic gastric neoplasm with a tan-white, fleshy serosal surface is shown. (a) Serosal aspect demonstrating an outwardly projecting mass arising from the muscularis propria. (b) Luminal aspect demonstrating submucosal protrusion with intact overlying gastric mucosa, consistent with a non-epithelial origin. Colored arrows denote key features: Red, tumor bulk; blue, intact mucosa/serosa; yellow, tumor origin at the muscularis propria. Ruler included for size reference.

**Figure 3:**
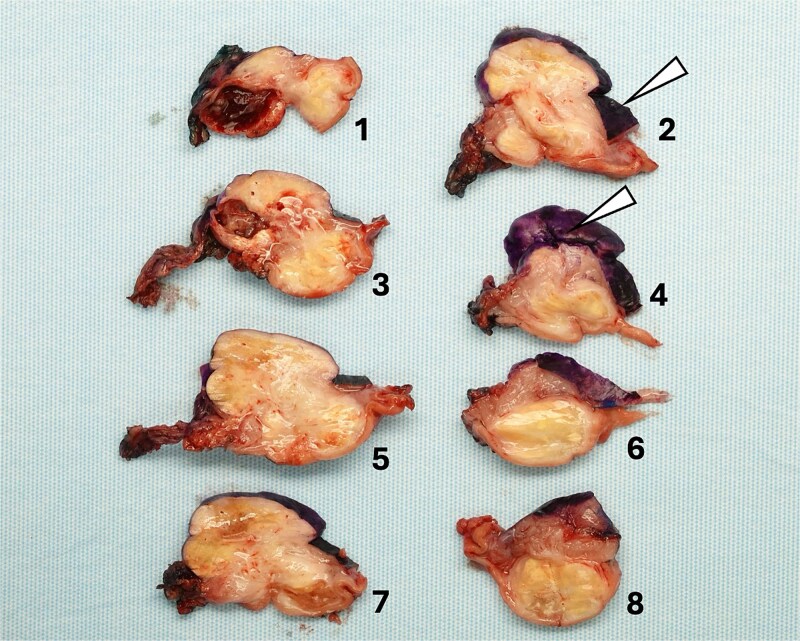
Serial gross sections of the resected gastric wedge specimen, Case 1. Cross-sections reveal a solid, tan-white, fleshy neoplastic mass with areas of multifocal hemorrhagic degeneration and focal necrosis. Peripheral surgical margin ink is applied for orientation and margin assessment (arrowheads). Sequential slices are numbered (1–8) for topographic mapping and histopathologic correlation; display arrangement is non-chronological relative to anatomic order.

The patient remained hemodynamically stable postoperatively. Analgesia was managed via a multimodal regimen. The patient demonstrated return of gastrointestinal function, advancing to a mechanical soft diet, and was discharged on postoperative day 5. Final histopathological assessment demonstrated positive proximal serosal margins, lymphovascular invasion, and malignant involvement of perigastric lymph nodes (Fig. 4). A multidisciplinary tumor board recommended adjuvant chemotherapy and radiation therapy.

**Figure 4:**
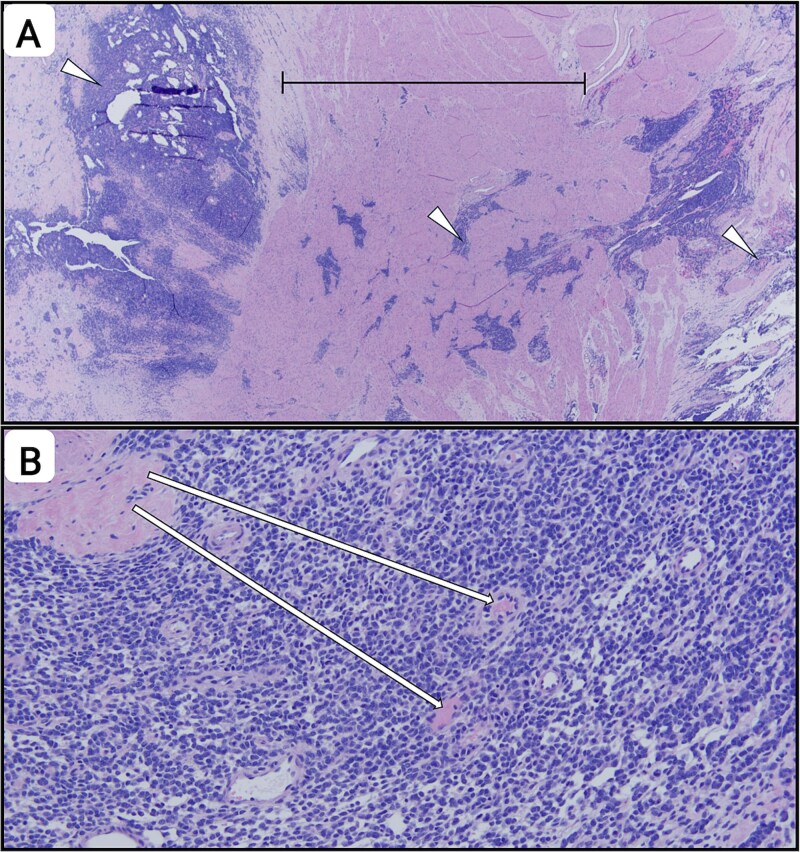
Photomicrographs (H&E stain) of the excised wedge gastrectomy specimen, Case 1, highlighting high-risk histopathological features. (a) Low-power magnification demonstrating transmural neoplastic infiltration traversing the full thickness of the gastric wall, extending from the serosal surface (left) through the muscularis propria and submucosa to the mucosa (right) (bracket), consistent with pathologic T4a staging. Representative foci of desmoplastic stroma with irregularly infiltrating tumor cell nests are indicated (arrowheads). (b) High-power magnification demonstrating lymphovascular invasion (LVI), characterized by cohesive neoplastic emboli within thin-walled, endothelial-lined vascular channels (arrows). The intraluminal tumor cell clusters are closely opposed to the endothelium, confirming intravascular tumor involvement, an independent predictor of nodal metastases and decreased survival.

### Case 2

A 12-year-old female presented with a 1-week prodrome of diffuse abdominal pain, dorsalgia, and cephalalgia. She was initially triaged in the emergency department, where single-view supine abdominal radiography suggested fecal impaction. However, her symptoms progressed over the following week to include worsening abdominal distension, persistent cephalalgia, diarrhea, emesis, and low-grade pyrexia, prompting repeat evaluation.

Initial hematologic analysis revealed a moderate anemia (Hb < 8.7 g/dl, Hct 28.0%) and a marked thrombocytosis (platelet count 726 × 10^9^/l). CECT of the chest, abdomen, and pelvis localized a large 9.6 cm × 11 cm intraperitoneal mass centered within the mesentery, with disseminated peritoneal sarcomatosis, omental caking, and high-volume ascites (Fig. 5). While a 3.6 cm ill-defined hypodensity was noted in hepatic segment VII, subsequent PET/CT confirmed intense hypermetabolic activity (SUV_max_) restricted to the primary mesenteric mass and peritoneal seedings, with the hepatic lesion remaining metabolically silent. Concurrent magnetic resonance imaging (MRI) of the abdomen further corroborated these findings.

**Figure 5:**
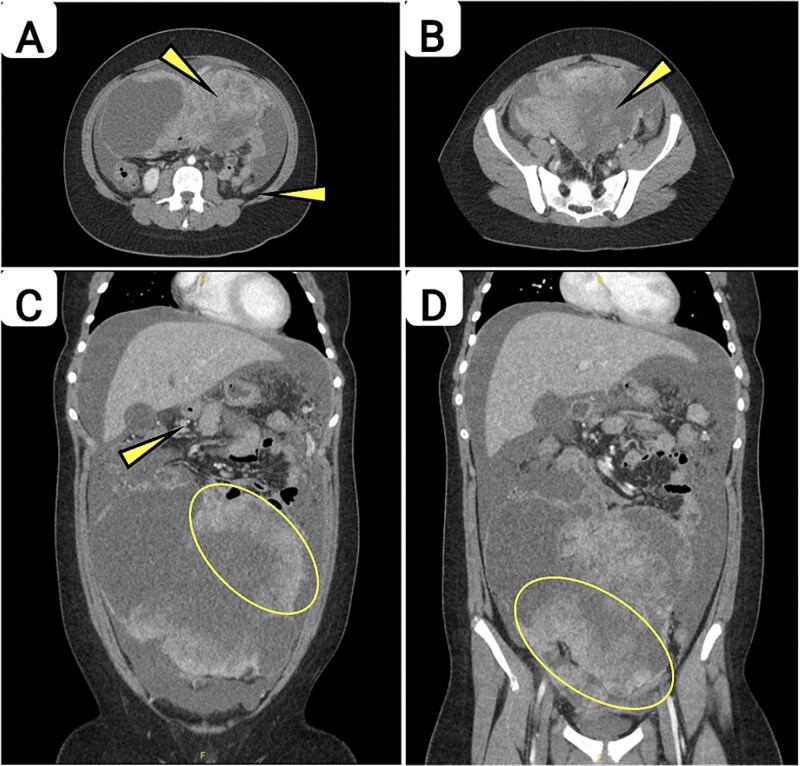
Baseline contrast-enhanced CT of the abdomen and pelvis acquired prior to initiation of systemic therapy, demonstrating extensive intra-abdominal and pelvic disease burden. (a) Axial CECT, upper abdomen: A voluminous, heterogeneously enhancing mesenteric mass (arrow) is identified, inducing significant mass effect with resultant displacement and compressive remodeling of adjacent enteric loops. Concomitant moderate-volume hypoattenuating intraperitoneal free fluid (arrow) is observed, consistent with malignant ascites in the appropriate clinical context. (b) Axial CECT, pelvis: A large, lobulated, heterogeneously enhancing soft tissue mass (arrow) occupies the central pelvic cavity, producing mass effect with cephalad and lateral displacement of adjacent pelvic viscera, including the urinary bladder and rectosigmoid colon. This finding is consistent with caudal extension of the dominant intra-abdominal tumor burden into the pelvic compartment. (c) Coronal CECT, abdomen, and pelvis: Diffuse peritoneal carcinomatosis is demonstrated. Circumferential infiltration and nodular thickening of the greater omentum (ellipse) are identified, consistent with omental caking. Multifocal soft tissue nodularity along the mesenteric folds and peritoneal reflections (arrow) is indicative of disseminated peritoneal sarcomatosis. Large-volume ascites is present, contributing to elevated intra-abdominal pressure and wide separation of bowel loops. (d) Coronal reformatted image: A comprehensive depiction of aggregate disease burden is provided, demonstrating a dominant, confluent abdominopelvic conglomerate mass (ellipse) extending contiguously from the mid-abdominal mesentery into the true pelvis. The composite imaging phenotype, comprising a primary mesenteric mass, diffuse omental caking, and large-volume ascites, constitutes a diagnostic triad of advanced-stage intra-abdominal malignancy at clinical presentation.

Following multidisciplinary consultation involving hematology/oncology and palliative care teams, diagnostic laparoscopy with concomitant peritoneal and omental biopsy, bone marrow (BM) aspiration, and mediport placement was performed. Histopathologic and molecular assays identified a non-osseous EWS harboring an *EWSR1-ERG* positive fusion variant. While peritoneal cytology was negative for malignancy, BM biopsy confirmed occult metastatic involvement, and omental biopsy revealed an infiltrative desmoplastic small round cell tumor (DSRCT) pattern consistent with extraosseous Ewing sarcoma, further delineating the extent of disseminated disease (Fig. 6).

**Figure 6:**
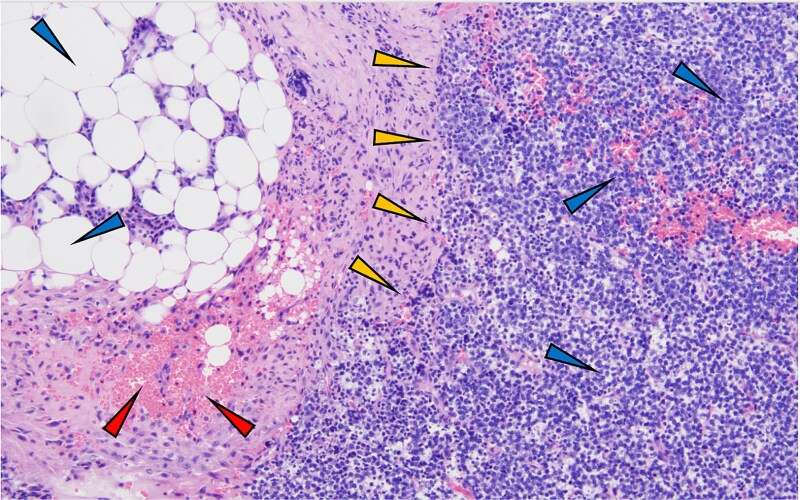
Omental biopsy (H&E preparation, 100× magnification) revealing an infiltrative DSRCT consistent with extraosseous Ewing sarcoma, Case 2. The photomicrograph demonstrates effacement of indigenous omental stroma by cohesive sheets of uniform cells exhibiting a markedly elevated nuclear-to-cytoplasmic (N:C) ratio, finely stippled basophilic chromatin, and attenuated eosinophilic cytoplasm. The neoplastic proliferation, occupying the right aspect of the field (blue arrowheads), directly abuts intact univacuolated mature adipocytes along the left (blue arrowheads). The transition is delineated by a desmoplastic fibrocollagenous band (gold arrowheads) containing hemorrhagic necrosis (red arrowheads), highlighting the aggressive architecture of the tumor as it supplants resident adipose parenchyma.

The patient commenced aggressive systemic chemotherapy during her initial hospitalization and was discharged on postoperative day 11. Her clinical course was punctuated by recurring hospitalizations for significant treatment-related morbidities, predominantly driven by profound chemotherapy-induced neutropenia manifesting as respiratory distress, skin and soft tissue infections, and multiple bacteremic episodes. Notably, the patient developed *Pseudomonas* bacteremia warranting emergent mediport explantation, alongside incision and drainage (I&D) of a left gluteal abscess. Despite these complications, chemotherapy was continued.

Four months post-induction, interval MRI demonstrated a robust partial response with marked reduction in tumor burden (Fig. 7). Following 8 months of systemic therapy, the patient underwent definitive surgical cytoreduction via a midline exploratory laparotomy.

**Figure 7:**
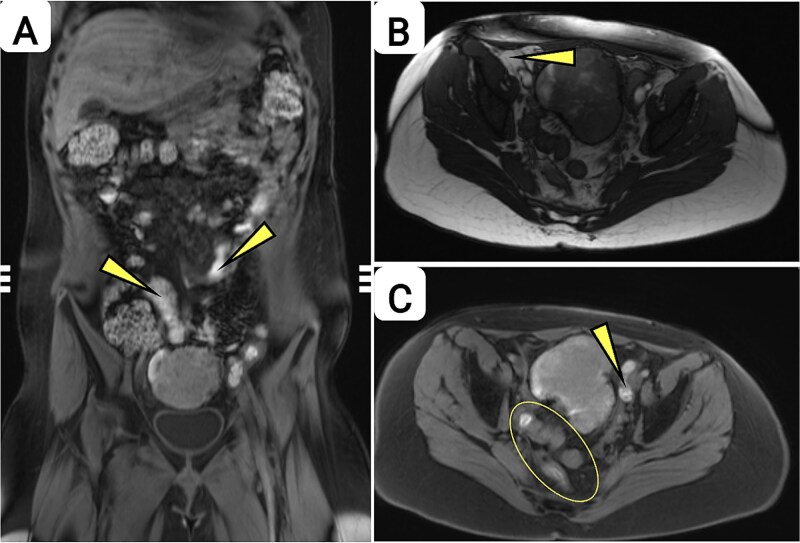
Post-chemotherapy MRI of the abdomen and pelvis demonstrating marked interval cytoreduction with residual multifocal abdominopelvic disease. (a) Coronal T2-weighted/post-contrast image: A substantial quantitative reduction in aggregate neoplastic burden is demonstrated relative to baseline imaging, characterized by significant interval diminution in the dimensions of the dominant intra-abdominal mass. Persistent, T2-hypointense multifocal peritoneal implants (arrows) remain distributed along the mesenteric and serosal surfaces, consistent with residual peritoneal sarcomatosis. A residual pelvic lesion is also identified at the inferior margin. (b) Axial image, upper pelvis: Residual mesenteric and peritoneal soft tissue thickening (arrow) is demonstrated, reflecting a marked longitudinal reduction in disease volume following administration of systemic cytotoxic therapy. The morphologic characteristics of the residual tissue are suggestive of a combination of viable tumor and post-treatment desmoplastic change. (c) Axial image, pelvis: A well-circumscribed residual pelvic mass (arrow) is identified, constituting the principal site of persistent macroscopic disease. Adjacent clustered nodular foci along the pelvic peritoneal reflections (ellipse) correlate with residual serosal implants. The composite imaging findings are indicative of a robust partial response (PR) as defined by RECIST version 1.1, with stable focal residual disease amenable to potential surgical consolidation.

Intraoperative findings revealed complete resolution of omental disease and a pelvic mass characterized by extensive treatment-effect necrosis. Residual disease was localized to a solitary peritoneal implant in the right upper quadrant and paired implants on the anterior uterine and rectal serosa. Due to dense visceral adhesions between the primary pelvic nidus and distal small ileum, an en bloc segmental enterectomy was performed with continuity restoration via a stapled side-to-side anastomosis, alongside an oversewing of the bladder resection margin to ensure mural integrity. All visible peritoneal implants were successfully cytoreduced, and intraoperative frozen section analysis revealed no viable tumor.

Following surgical convalescence, she transitioned to an outpatient multimodal regimen comprising adjuvant chemotherapy and consolidative radiotherapy. Her course was further encumbered by late-phase toxicities, including non-occlusive portal vein thrombosis, refractory ascites, and pulmonary consolidation secondary to pneumonia. Given the burden of cumulative treatment-related toxicity, a joint decision led to the cessation of active treatment ~9 months post-surgical cytoreduction. Longitudinal post-treatment surveillance via serial MRI confirmed a complete radiographic response. Continued biennial imaging over a 5-year period has demonstrated no recurrence. The patient remains disease-free and asymptomatic to date.

## Discussion

From a cytogenetic perspective, Ewing sarcomas have been molecularly delineated as fusion-driven transcriptional malignancies [3, 11]. The vast majority of cases (~85%–90%) involve a balanced translocation of the *EWSR1* gene to a member of the ETS-family transcription factor group [3, 12]. While the *EWSR1::FLI1* fusion predominates, the *EWSR1::ERG* variant observed in the second patient represents a distinct molecular subset, accounting for ~10%–15% of cases [3, 11, 12]. These chimeric proteins function as aberrant neo-transcription factors, inducing widespread epigenetic reprogramming and chromatin remodeling by recruiting complexes such as BAF (SWI/SNF), MLL, and p300 to create de novo enhancers at GGAA microsatellites [3, 13, 14]. This molecular driver sustains a primitive, poorly differentiated cellular phenotype while maintaining autonomous proliferative signaling, as EWS-FLI1 impairs cellular differentiation through miRNA dysregulation (e.g. miR-145 suppression) and maintains tumor cell plasticity [15]. Given the morphological ambiguity of “small round blue cell tumors,” molecular confirmation via FISH or NGS is imperative to distinguish visceral ES from other clinicopathological mimics like rhabdomyosarcoma or DSRCT, as these entities share overlapping morphology but harbor distinct translocations (e.g. *PAX3::FOXO1* in alveolar rhabdomyosarcoma, *EWSR1::WT1* in DSRCT) [16–18].

The aforementioned cases underscore the danger of anchoring bias in pediatric gastrointestinal pathology, where visceral Ewing sarcomas can masquerade as benign etiologies. In the gastric case, severe microcytic anemia preceded overt symptoms and was misattributed to nutritional deficiency; in the omental case, distention was initially thought to reflect functional constipation. The correct diagnosis became apparent only when the clinical trajectory diverged from benign expectations. For the pediatric surgeon, persistent systemic findings—such as refractory anemia or escalating pyrexia—and progressive local symptoms should necessitate early cross-sectional imaging and tissue sampling rather than repeated conservative interventions.

The choice of diagnostic approach should be guided by the tumor’s anatomic compartment. For intramural gastric lesions, EUS with core biopsy provides a high-yield, minimally invasive option. Conversely, when omental sarcomatosis is suspected, diagnostic laparoscopy offers the combined advantage of obtaining representative tissue and performing macro-staging. In both patients, neoadjuvant chemotherapy substantially reshaped the surgical landscape. By reducing tumor burden and promoting a peripheral “pseudocapsule,” systemic therapy enabled function-preserving R0 resections—a partial gastrectomy and en bloc enterectomy—rather than morbid, purely extirpative surgery. This supports a treatment paradigm where surgical timing is guided by interval biological response rather than initial anatomical presentation alone.

Lastly, these cases illustrate a critical radiographic-pathological dissonance: morphological response on cross-sectional imaging is an unreliable proxy for true biological clearance. In the gastric patient, despite favorable radiographic regression, the excised specimen retained high-risk histopathological features, connoting persistent biological volatility. Conversely, the omental case showed extensive treatment-effect necrosis and achieved durable, long-term remission, even though toxicity required premature discontinuation of chemotherapy. In practice, these findings validate an adaptive, multimodal framework where molecular profiling defines the diagnosis, while the neoadjuvant phase functions as an in vivo test of chemosensitivity. Ultimately, achieving lasting remission hinges on prioritizing pathological viability over radiographic tumor volume when assessing the effectiveness of therapy.

## Conclusion

This case series contributes to the sparse pediatric literature on primary visceral EES by presenting two unusual primary sites: the gastric wall and omentum. Both instances illustrate how these tumors can elude early recognition due to nonspecific symptoms and plausible alternative explanations, leading to considerable diagnostic latency. The series supports a low-threshold for cross-sectional imaging and tissue biopsy in pediatric patients exhibiting refractory anemia, progressive abdominal distention, or atypical clinical courses. Additionally, these cases emphasize the importance of a multimodal treatment strategy–integrating molecular diagnostics, neoadjuvant systemic chemotherapy, and individualized organ-sparing surgical resection. Notably, the observed discrepancy between radiographic response and pathological risk suggests that therapeutic pivots should be grounded in tumor biology rather than isolated imaging metrics.
